# Supplementary material for: Transposon invasion of primate genomes shaped human inflammatory enhancers and susceptibility to inflammatory diseases
Source: Nat Commun. 2025 Nov 3;16:9674. doi: 10.1038/s41467-025-64690-7 (PMC12583588; doi:10.1038/s41467-025-64690-7)
Supplement: Supplementary file 1 — Supplementary Information [file 41467_2025_64690_MOESM1_ESM.pdf]

Primate-specific transposable elements shape the evolution of inflammation-related enhancers

Mengliang Ye, Maxime Rotival, Sebastian Amigorena, Elina Zueva

Corresponding author: ella.zueva@curie.fr

The PDF file includes:

Supplementary Figures 1-8.

Each Supplementary Figure is accompanied by its corresponding data in the Source Data file.

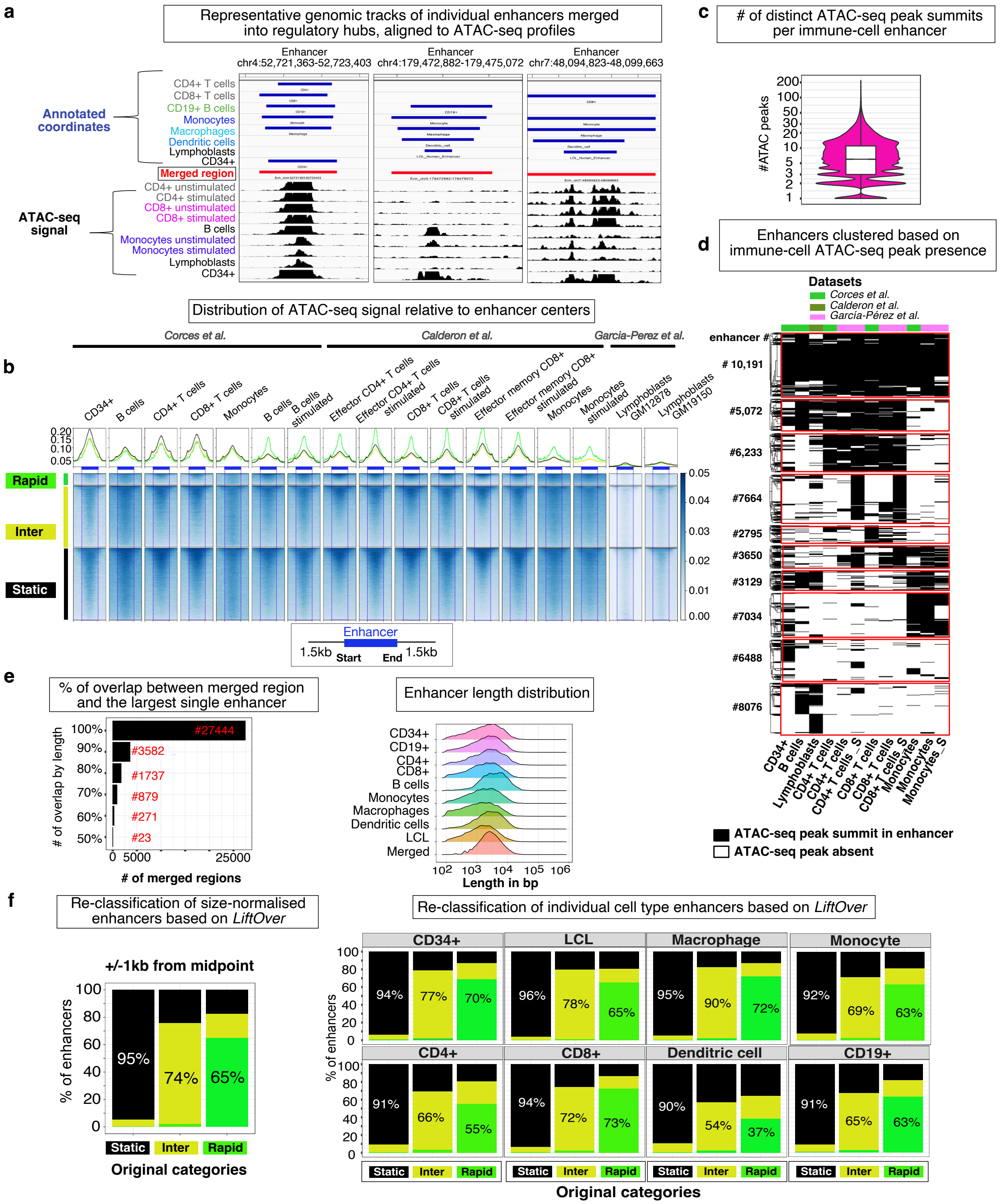

Supplementary Figure 1. Characterization of pan-immune cell enhancers created from pooling individual cell type annotations. **a**, Genome browser view of merged enhancer coordinates. Annotated enhancer regions from individual cell types are shown, with the final merged region highlighted in red, alongside representative ATAC-seq tracks from distinct immune cell populations. **b**, Aggregate ATAC-seq signal centered on enhancer midpoints, reflecting chromatin accessibility across cell types. **c**, Number of non-redundant ATAC-seq peak summits (pooled across immune populations) per merged enhancer. **d**, Binary k-mean clustering map showing the presence (black) or absence (white) of ATAC-seq summits from individual immune cell types within each immune-cell enhancer. Number of enhancers per cluster is shown. “S” for stimulated **e**, **Left**; Percentage of overlap between merged enhancers and the largest contributing enhancer from individual cell types. **Right**: Size distributions of original cell-type-specific enhancers and the final merged pan-immune enhancer set. **f**, Re-classification of enhancers based on LiftOver conversion to the macaque and chimpanzee genomes. **Left**: Enhancers normalized to 2 kb (±1 kb from midpoint). **Right**: Individual cell-type enhancers. Color bars below indicate original LiftOver-based classification categories of not size-normalised regions.

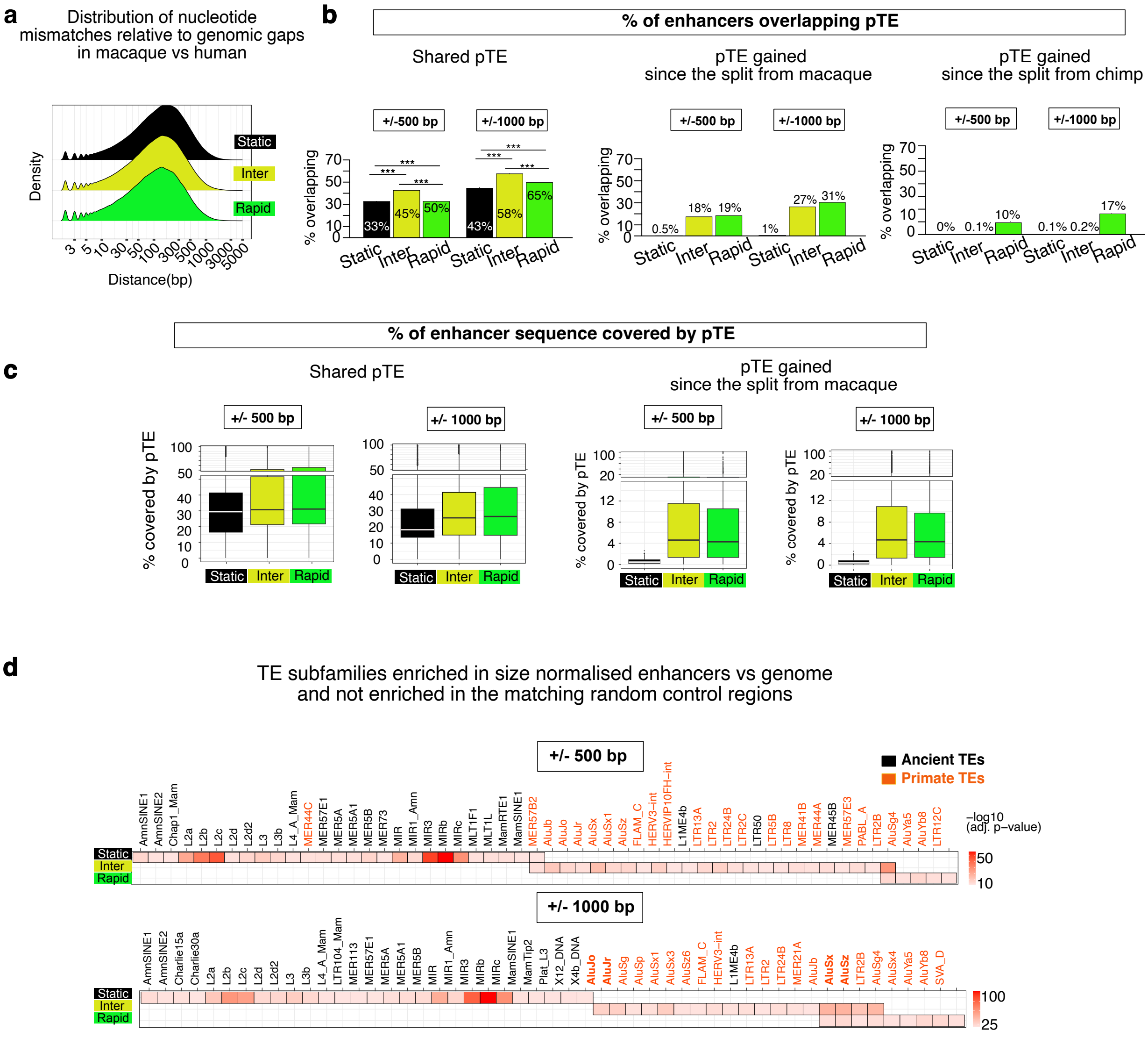

Supplementary Figure 2. Additional controls for Fig.1

a, Distribution of single-nucleotide substitution distances from genomic alignment gaps in human/macaque comparisons. **b and c,** Quantification of pTE content in enhancers, normalised to ±500 bp and ±1,000 bp from the enhancer midpoint. **b**, Proportion of enhancers overlapping (by ≥ 10bp) with pTEs that are either present (shared; **left**), or absent (gained) based on coincidence with genomic gaps in macaque (**middle**) and chimpanzee (**right**) relative to human. ***P ≤ 0.001. **c**, Fraction of enhancer length covered by pTEs, considering shared pTEs (**left**) or only those absent in macaque (**right**). **d**, TE subfamilies most significantly enriched in size normalised enhancers (hypergeometric enrichment test adjusted P < 1e-7, fold change ≥2, n ≥ 10), but not enriched in matched non-enhancer regions shuffled 1,000 times.

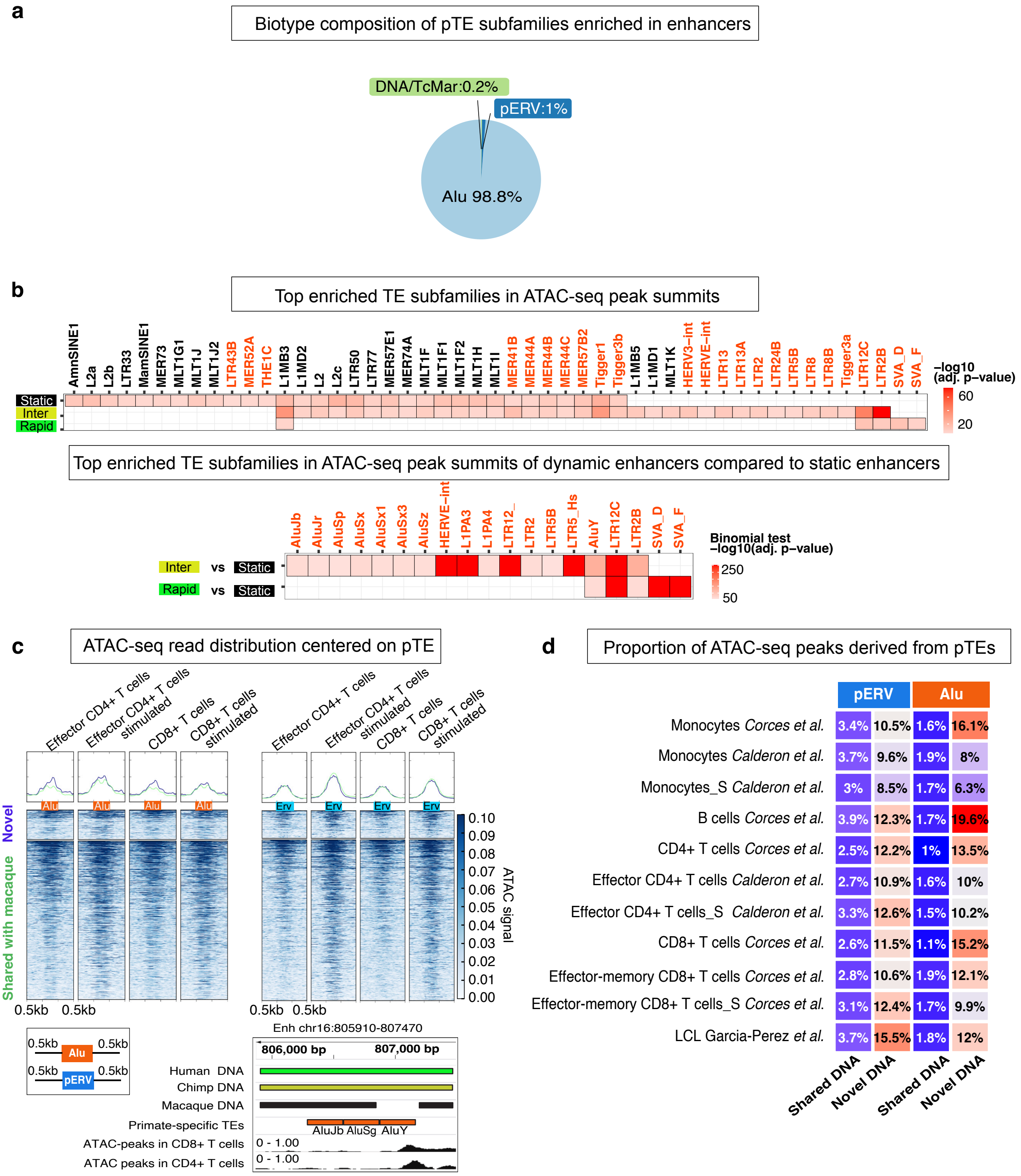

Extended Data Figure 3. Dominance and accessibility of Alu and pERV elements in enhancers.

a, Biotype composition of pTEs from enriched subfamilies (grouped by phylogeny) within enhancers. **b**, Enrichment of TE subfamilies at ATAC-seq peak summits across immune cell types. **Top**: Enrichment relative to the genome (adjusted hypergeometric test, $P < 10^{-7}$; ≥10 copies; fold-change ≥10). Bottom: Enrichment at summits of dynamic versus static enhancers (adjusted binomial test, $P < 10^{-7}$; ≥10 copies). **c**, Aggregate ATAC-seq signal from representative immune populations centered on Alu and pERV elements occupying ≥50% of peak length, stratified by elements shared with macaque or gained post-split. **Bottom**: Genome browser view of an enhancer containing a novel AluY insertion at the ATAC-seq peak summit. **d**, Proportion of pTE-derived sequence within ATAC-seq peaks mapping to either shared (≥50% aligns to RheMac10) or novel (≥50% aligns to macaque genomic gaps) DNA.

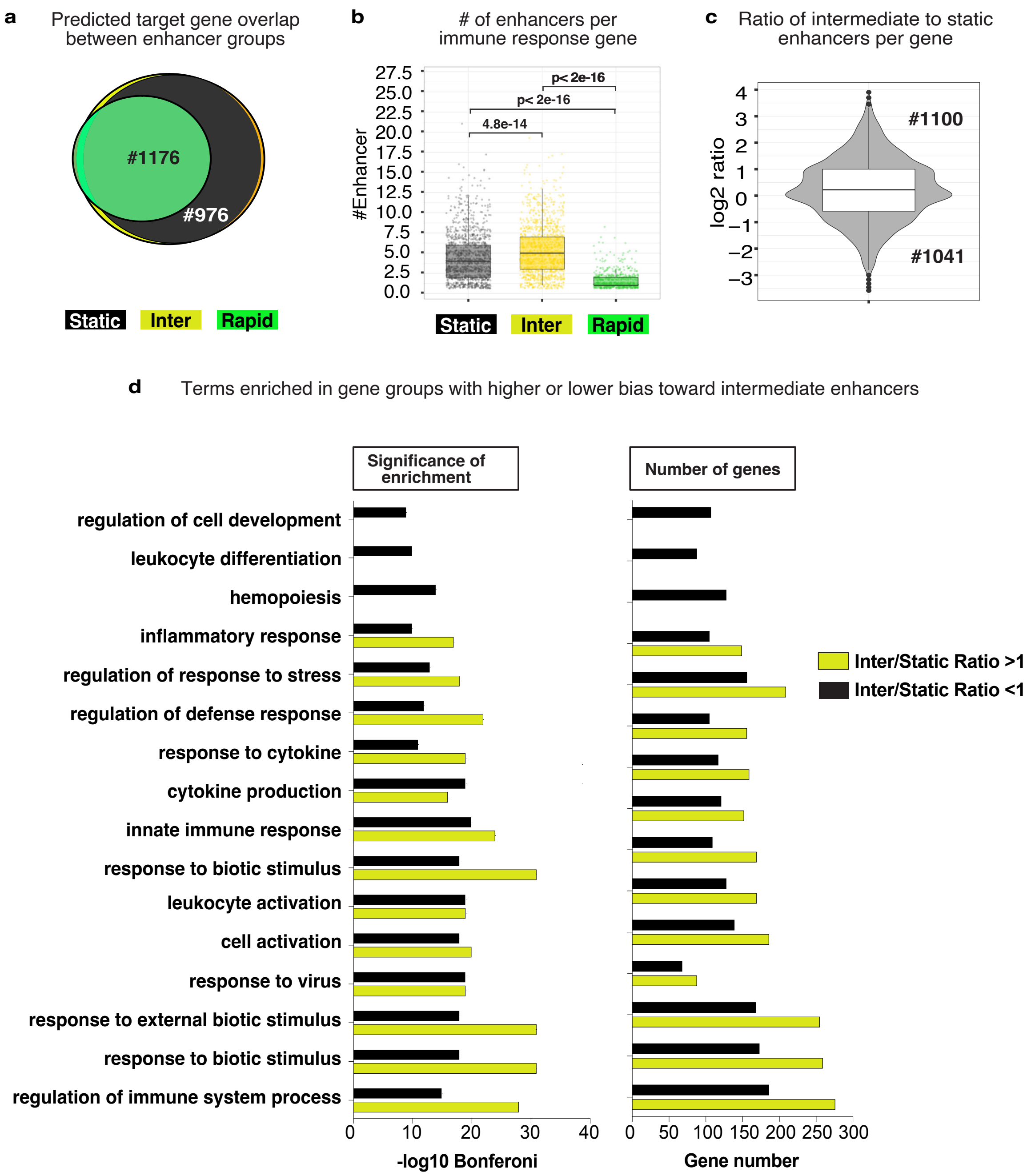

Extended Data Figure 4. Distinct functionality of enhancer groups.

a, Overlap between genes assigned to distinct enhancer groups based on activity-by-contact (ABC)-predicted enhancer–gene interactions. Only genes upregulated following 4 hours stimulation of human blood cells with infectious agents (from Hawash et al.[1]) are included. **b,** Number of enhancers per immune-response genes, as defined by ABC algorithm. **c,** Distribution of genes with different intermediate-to-static enhancer ratios; gene counts above and below a ratio of one are indicated. **d,** Functional terms enriched in gene groups with a higher or lower bias toward intermediate enhancers, accessed using Toppgene Suite.

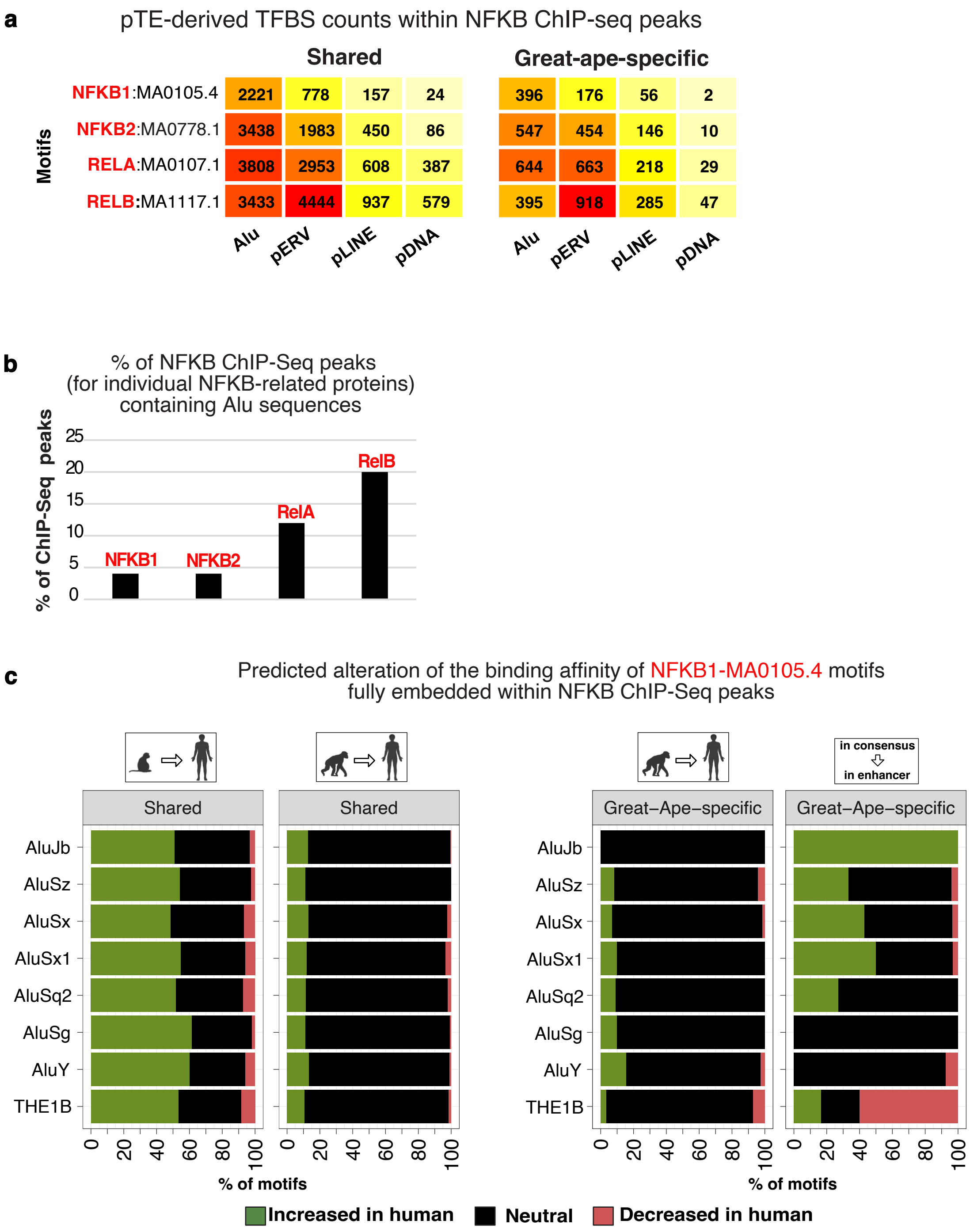

Extended Data Figure 5. TFBS and pTEs in ChIP-seq peaks.

a, Number of NF-κB motifs in human genome, shared or not with the macaque genome, fully embedded within NF-κB ChIPseq peaks, categorized by distinct pTE biotypes. **b,** Proportion of ChIP-seq peaks for the distinct NF-κB proteins overlapping (by ≥10bp) with Alus carrying NF-κB motifs. **c,** TFBS embedded within NF-κB CHIP-Seq peaks **Left panels**: Binding affinity differences (Δ) for pTE-derived NF-κB1 motifs (MA00105.4) shared between human and macaque/chimpanzee genomes, predicted using TFBStools. **Right panels:** Δ for great-ape-specific motifs relative to chimpanzee orthologs and human consensus sequences.

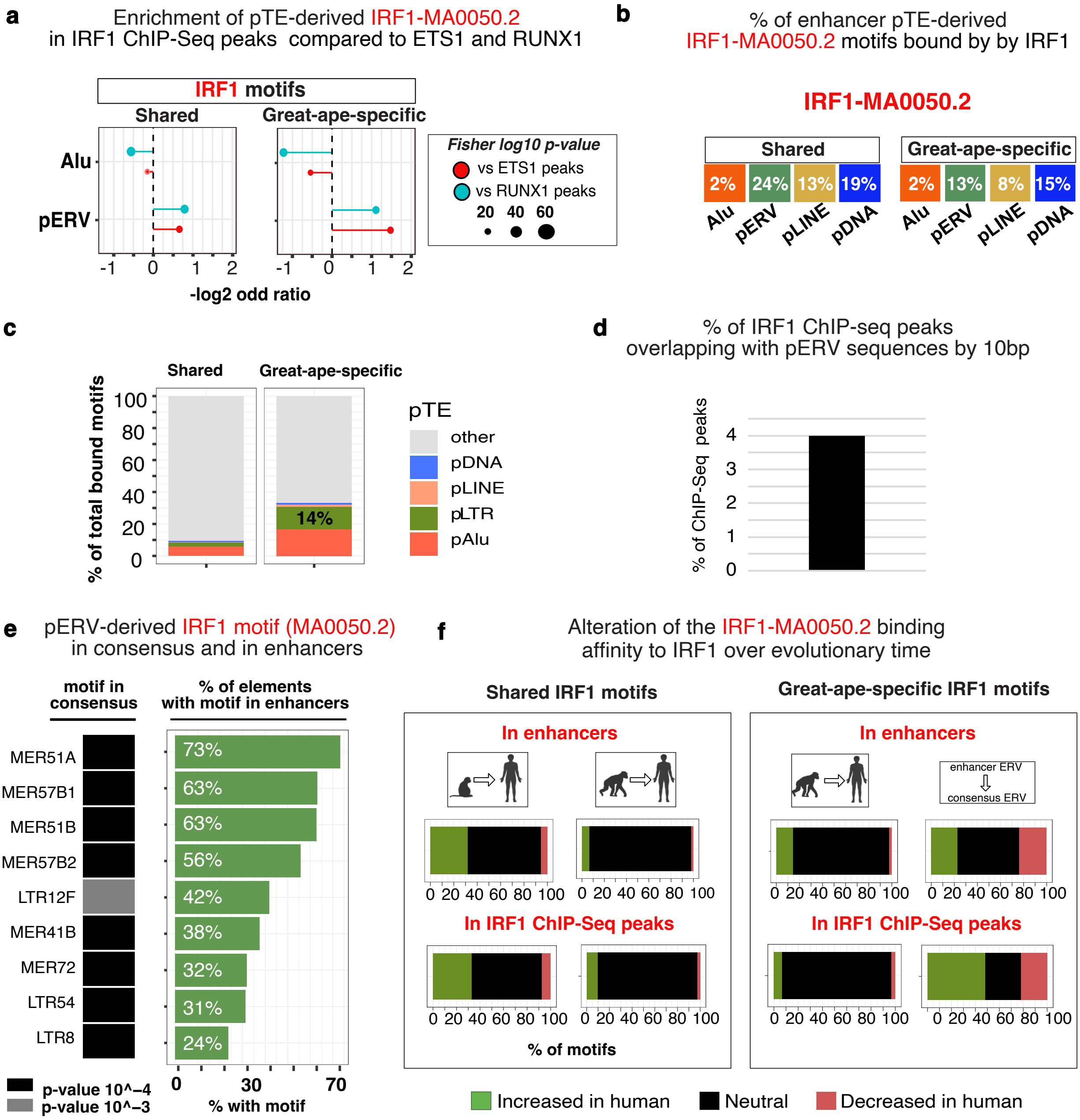

Supplementary Figure 6. Evolution and functionality of the pTE-derived IRF1 motifs. **a,** Enrichment or depletion tested for Alu- or pERV-derived IRF1 motifs embedded within IRF1 ChIP-seq peaks, relative to the same motifs within ETS1 and RUNX1 ChIP-seq peaks. **b**, Fraction of IRF1-bound (by ChIP-seq) IRF1 (MA0050.2) motifs from total within distinct pTE types. **c**, Contribution of different enhancer sequences to IRF1-bound IRF1 motifs, split by shared and great-ape-specific motifs; pTEs are highlighted. **d**, Proportion of IRF1 CHIP-seq peaks overlapping (by ≥10bp) with pERVs carrying IRF1 motifs. **e**, **Left:** IRF1 motifs identified in consensus sequences of pTE subfamilies that are most abundant in IRF1 ChIP-seq peaks (>10 copies). Black: identified by FIMO screening of JASPAR2022 PWMs with stringent match ($P < 0.0001$); Grey: identified with permissive match ($P < 0.001$) **f, Left panels:** Binding affinity differences (Δ) for shared pERV-derived IRF1 motifs (all in enhancers and a cohort embedded within IRF1 CHIP-Seq peaks) between human and macaque/chimpanzee genomes, predicted using TFBStools. **Right panels:** Δ for great-ape-specific motifs (all in enhancers and IRF1-bound) relative to chimpanzee orthologs and human consensus sequences.

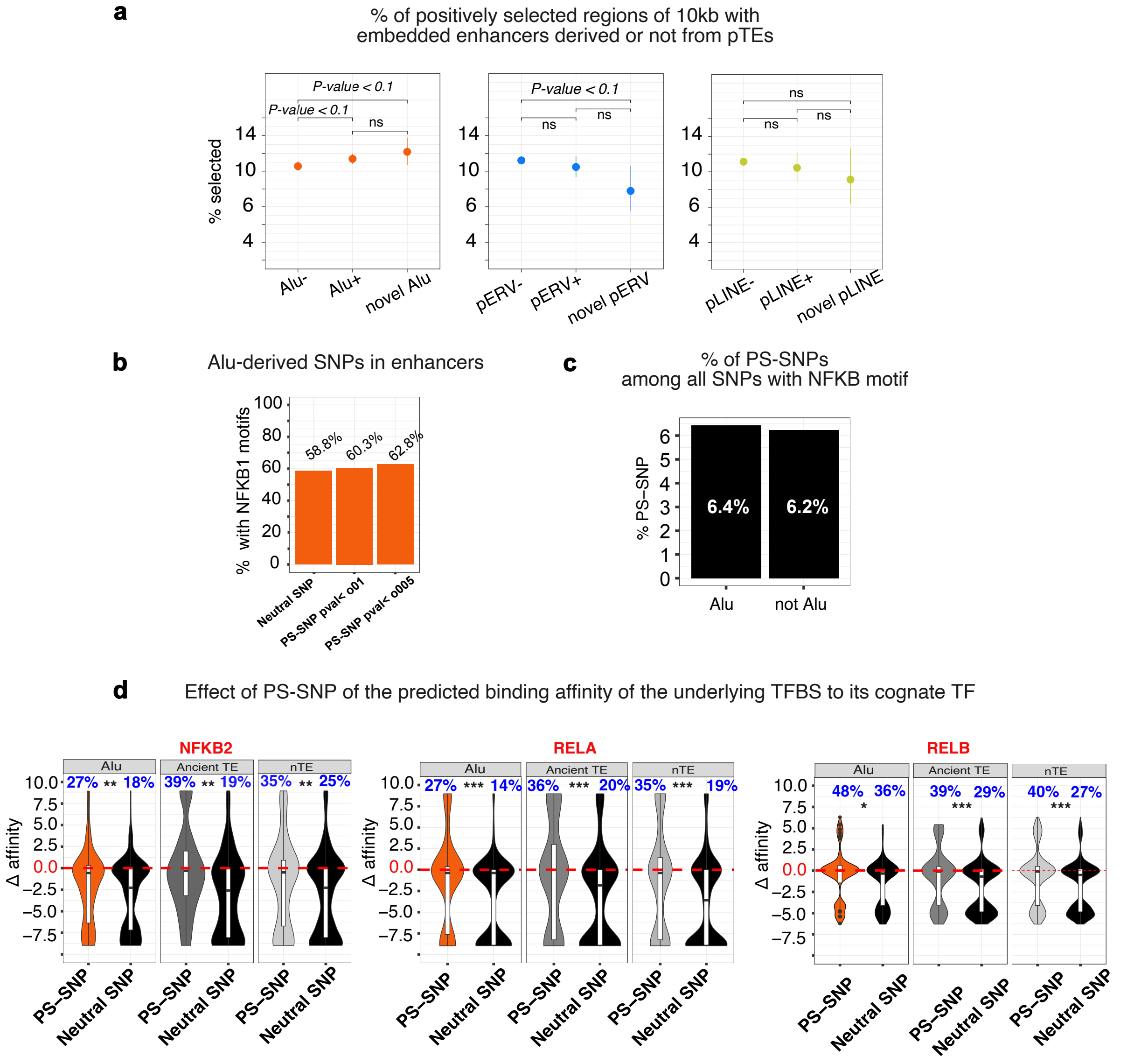

Supplementary Figure 7. Characterization of Alu and NFKB motif-carrying enhancers with the evidence of positive selection

a, Proportion of enhancers under positive selection measured within ±5 kb from their midpoints. Compared are enhancers lacking Alus, pERVs, or pLINEs (either shared with or absent in the macaque genome, RheMac10). Dots show the percentage of positively selected enhancers (5% locus-level FDR) in each group; Error bars indicate 95% confidence intervals from an exact binomial test. Pvalues were obtained using pairwise two-sided Fisher's exact test adjusted by the Benjamini–Hochberg method. **b**, Proportion of Alu-derived NF-κB-MA0105.4 motifs overlapping enhancer PS-SNPs (*P*>0.01) across different significance ranges, as well as neutral SNPs (*P*>0.5), frequency-matched in the same population, relative to the total number of NF-κB1-MA0105.4 motifs in each category. **c**, Proportion of PS-SNPs among all SNPs overlapping NF-κB1-MA0105.4 motif, stratified by Alu-derived or non-TE-derived locations. **d**, Distribution of NF-κB-related motifs' binding affinity changes (Δ derived allele vs. ancestral) estimated using TFBStools for PS-SNPs and neutral SNPs, both located in enhancers, stratified by sequence origin. Differences in the proportion of TFBS with positive Δ between groups were tested with a two-sided χ^2 test. *** P < 0.001, ** P < 0.01, * P < 0.05.

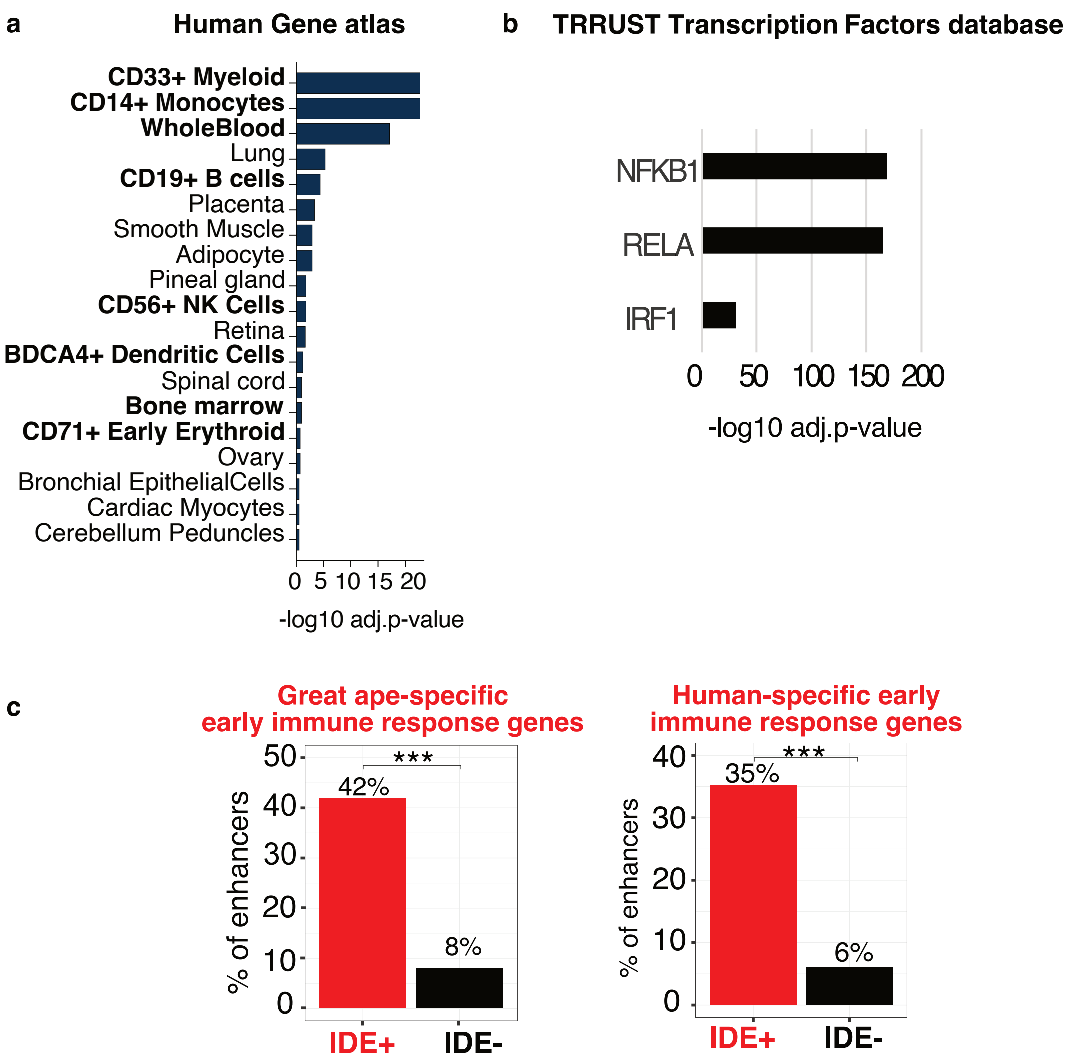

Supplementary Figure 8. Characterization of enhancers potentially associated with inflammatory diseases.

a, Enrichment of inflammatory disease signature genes (shared by 30% of various conditions), accessed using the Human Cell Atlas tool in the EnrichR hub. **b**, TF target enrichment of the inflammatory disease signature, accessed using the TRRUST Transcription Factors 2019 tool within the EnrichR hub. **c**, Proportion of enhancers associated with genes (by ABC maps) that show a stronger early transcriptional immune response in great apes (including humans) compared to macaques and in humans compared to great apes. Statistical comparisons were performed using pairwise two-sided χ^2 tests. *** $P < 0.001$.
