# Supplementary material for: Transposon invasion of primate genomes shaped human inflammatory enhancers and susceptibility to inflammatory diseases
Source: Nat Commun. 2025 Nov 3;16:9674. doi: 10.1038/s41467-025-64690-7 (PMC12583588; doi:10.1038/s41467-025-64690-7)
Supplement: Supplementary file 2 — Description of Additional Supplementary Files [file 41467_2025_64690_MOESM2_ESM.pdf]

Description of Additional Supplementary Files

File Name: Supplementary Data 1
Description: Public sources of annotated immune-cell enhancers and ATAC-Seq data for constructing a catalog of active immune-cell enhancers

File Name: Supplementary Data 2
Description: Putative human pan-immune-cell enhancers stratified according to the LiftOver alignability to the macaque and chimpanzee genomes

File Name: Supplementary Data 3
Description: Statistics of the enrichment of TE subfamilies within distinct enhancer groups

File Name: Supplementary Data 4
Description: Quantification of TFBS in enhancers

File Name: Supplementary Data 5
Description: Enhancer gene targets predicted by the activity-by-contanct maps from Nasser et al.

File Name: Supplementary Data 6
Description: CHIP-Seq datasets used in this study

File Name: Supplementary Data 7
Description: SNPs and enhancers under positive selection in human populations

File Name: Supplementary Data 8
Description: Enhancers potentially associated with inflammatory and autoimmune disorders

File Name: Supplementary Data 9
Description: Alus within immune-cell enhancers, whose depletion in Liang et al. impacts gene expression
